# Recentralization within decentralization: County hospital autonomy under devolution in Kenya

**DOI:** 10.1371/journal.pone.0182440

**Published:** 2017-08-03

**Authors:** Edwine W. Barasa, Anthony M. Manyara, Sassy Molyneux, Benjamin Tsofa

**Affiliations:** 1 KEMRI Centre for Geographic Medicine Research–Coast, and Wellcome Trust Research Programme, Nairobi, Kenya; 2 Centre for Tropical Medicine, University of Oxford, Oxford, United Kingdom; Kenya Medical Research Institute - Wellcome Trust Research Programme, KENYA

## Abstract

**Background:**

In 2013, Kenya transitioned into a devolved system of government with a central government and 47 semi-autonomous county governments. In this paper, we report early experiences of devolution in the Kenyan health sector, with a focus on public county hospitals. Specifically, we examine changes in hospital autonomy as a result of devolution, and how these have affected hospital functioning.

**Methods:**

We used a qualitative case study approach to examine the level of autonomy that hospitals had over key management functions and how this had affected hospital functioning in three county hospitals in coastal Kenya. We collected data by in-depth interviews of county health managers and hospital managers in the case study hospitals (n = 21). We adopted the framework proposed by Chawla et al (1995) to examine the autonomy that hospitals had over five management domains (strategic management, finance, procurement, human resource, and administration), and how these influenced hospital functioning.

**Findings:**

Devolution had resulted in a substantial reduction in the autonomy of county hospitals over the five key functions examined. This resulted in weakened hospital management and leadership, reduced community participation in hospital affairs, compromised quality of services, reduced motivation among hospital staff, non-alignment of county and hospital priorities, staff insubordination, and compromised quality of care.

**Conclusion:**

Increasing the autonomy of county hospitals in Kenya will improve their functioning. County governments should develop legislation that give hospitals greater control over resources and key management functions.

## Background

Decentralization has been widely implemented in low and middle income countries as part of broader economic and political reforms [1,2]. Decentralization typically takes either or a combination of the following four forms [3]: *delegation*, where authority is transferred to semi-autonomous agencies that carry out functions that were previously carried out by government ministries; *deconcentration*, where authority is shifted from national structures to sub-national offices of the same national structures; *devolution*, where authority is shifted from national structures to structures within autonomous or semi-autonomous local governments; and *Privatization* where, ownership changes from public to private entities.

Kenya has a long history of decentralization dating back to the early 1980’s when the government published the district focus for rural development (DFRD) strategy in 1983 [4]. The DFRD policy identified the district as the most basic and effective unit for planning, development, and delivery of public services in Kenya [5]. The district was established as the basic level responsible for operational tasks with relatively limited strategic functions [5]. This was therefore a *deconcentration* type decentralization reform. Within the health sector, the district health system, which was managed by the district health management teams (DHMTs), was the focal point [6]. The core function of the DHMTs was to oversee all health sector activities within the districts and included the management and supervision of district hospitals and rural health facilities (sub-district hospitals, health centers, and dispensaries) [6]. With the coming into effect of a new constitution after the March 2013 general election, Kenya transitioned into a devolved system of government with a central government and 47 semi-autonomous county governments [7]. Under this new governance structure, the health system is structured such that the central Ministry of Health has retained policy making and regulatory roles while responsibilities such as allocation and managing health care resources and service provision have been transferred to county health systems [8]. The public healthcare delivery system is now organized into four tiers, namely community, primary care, county referral and national referral. Community health services include all community based demand creation activities that are guided by the MOH community strategy [9,10]. Primary healthcare include services provided by public and private maternity homes, health centers and dispensaries. County referral services include first level referral hospitals that are managed by a given county. National referral services comprise of (formerly) provincial and national level facilities, where tertiary referral services are provided.

Within the health sector, decentralization reform has been promoted as a means to improving health sector performance through, among others, improving the efficiency, responsiveness and local accountability of health systems [1,3,11]. Among its many promises, decentralisation aims to bring services closer to people [12,13], improve access to services,[3,12] allow for community participation [14,15], improve employee morale and turnover [16] and place particular emphasis on improving quality, access and equity, empowering local governments and increasing innovation and efficiency [12,13]. This reflects the Alma Ata Declaration in 1979 which highlighted the importance of bringing healthcare and decision-making as close as possible to where people live and work [17]. However, the effects of decentralization in the health sector have been mixed [2,15,16,18]. Decentralization reforms are highly complex and challenging to implement. As a result, the intended benefits may not always be achieved [12].

In this paper, we report early experiences of devolution in the Kenyan health sector, with a focus on public county hospitals. Specifically, we examine changes in hospital autonomy as a result of devolution, and how these have affected hospital functioning. Hospital autonomy refers to the reduction in direct government control over public hospitals, and a shift of the day-to-day decision making from the hierarchy to the hospital management team [19]. Hospitals are important to LMC health systems not only because they consume a significant proportion of health sector resources, but also because they are key platforms for the delivery of key curative interventions [20]. Increased hospital autonomy has the potential to result in, among other things, greater efficiency, improved quality of services, expanded accountability, increased understanding in communities about how hospitals operate and serve the communities, and improved equity in distribution of the services provided [21,22]. Decentralization reforms can significantly impact on the autonomy of hospitals [23]. Understanding how hospital autonomy has shifted within the context of devolution in Kenya, and how this has in turn influenced hospital functioning is therefore a critical question.

## Methods

### Study design

We used a qualitative case study approach, with data collected though in-depth interviews. A case study has been defined by Yin (2003) as "an empirical inquiry that investigates a contemporary phenomenon within its real life context" (p.13). In a case study, a phenomenon is examined and analysed in detail and depth using research tools that are most appropriate to the nature of the inquiry (Flyvbjerg, 2006). Kilifi county in coastal Kenya was purposefully selected as the case. Within Kilifi county, we conducted the study in 3 public county hospitals. The selection of Kilifi county was informed by two reasons. First, our research institution is located in Kilifi County and has long standing relationship working with and conducting research in the region. It was therefore administratively and logistically convenient to conduct the study in the county. Second, this study is part of a larger DFID funded study examining governance and accountability at the county health system level within the context of devolution in Kenya. The study employs a “learning site approach” where researchers embed themselves in the county health system, and collaboratively work with health system actors (managers, practitioners, policy makers) to “learn” the system; identify problems, formulate research questions and explore them and propose solutions. Learning sites allow for co-production between researchers and managers of insights and knowledge about health systems and decision-making practices [24]. Table 1 presents the characteristics of Kilifi county, while Table 2 presents the characteristics of the 3 county hospitals. To maintain confidentiality, and minimize the potential identification and victimization of study participants, the selected hospitals will only be identified as Hospital A, Hospital B, and Hospital C.

**Table 1:** Key demographic and health care information on kilifi county.

INDICATOR	KILIFI COUNTY
Total population	1,296,510
Male	625,658
Female	670,852
Under age 5	210,035
Under age 1	45,378
**HEALTH FACILITIES**	
Public	107
Non-governmental	9
Faith based	14
Private	127
**HEALTH PERSONNEL–PUBLIC HOSPITALS**	
Nurses (per 100,000 people)	30
Doctor (per 100,000 people)	4
Clinical officers (per 100,000 people)	17
**HEALTH FINANCING**	
Total government health spending (Per capita, KES)	1,433
National health insurance fund (NHIF) coverage in the county (% of county population)	24

Source: http://www.healthpolicyproject.com/pubs/291/Kilifi County

**Table 2:** Key characteristics of study hospitals.

Characteristic	Hospital A		Hospital B		Hospital C	
	Fiscal year 2013/14	Fiscal year 2014/15	Fiscal year 2013/14	Fiscal year 2014/15	Fiscal year 2013/14	Fiscal year 2014/15
Annual number of outpatient visits	27,006	37,161	68,722	97,307	45,105	50,170
Annual number of inpatient admissions	5,211	7,250	7,913	8,756	10,850	12,500
Number of inpatient beds	68		200		196	
Number of doctors	21		53		19	
Number of nurses	54		116		110	
Number of clinical officer	17		32		14	
Total number of permanent staff	171		129		599	
Total number of contracted staff	79		30		50	

Source: Health Records and Personnel Departments

### Data collection procedures

Since autonomy involves decision making in management, hospital decision makers in various levels were purposely selected for in-depth interviews. This included senior level and middle level hospital managers. Given that we were exploring autonomy in the context of devolution, we included 2 senior managers from the department of health services of Kilifi county government. A purposive sampling approach was used to select respondents for the in-depth interviews. A total of 21 in-depth interviews were conducted. Table 3 outlines the characteristics of interview participants.

**Table 3:** Distribution and roles of study participants.

Participant role	Hospital A	Hospital B	Hospital C	County Government
Senior managers	4	4	4	2
Middle level managers	2	2	4	
Total	6	6	7	2

We conducted interviews with selected participants after obtaining written informed consent for interviews and audio recording. We audio recorded interviews and supplemented this with note taking. Each of the interviews were face to face, took between 30–45 minutes, and were conducted within the study hospital premises.

### Conceptual framework

We adapted the framework proposed by Chawla and colleagues (1996) to examine hospital autonomy. This framework proposes that hospital autonomy can be unpacked into the level of control and influence that hospitals have over two key domains namely the health domain and the hospital domain [25]. The health domain refers to activities that are typically directed by the national governments, with limited control or influence by hospitals [25]. These include activities such as national goal setting, role definition and regulatory and policy formulation. The hospital domain refers to functions within the hospitals sphere of influence. Five key functions are included under this namely, 1) hospital administration, 2) financial management, 3) procurement, 4) human resource management, and 5) strategic management [25]. We focused our analysis on the hospital domain given that, within the devolution setting in Kenya, national level activities had limited or no role in hospital management. Rather, the roles identified in the hospital domain where more relevant to county hospitals in Kenya.

### Data management and analysis

We transcribed all interviews into word documents, and transferred them to NVIVO version 10 for coding. We analyzed the data using a framework approach. Framework analysis is a process that involves identifying connections between the data collected and a pre-determined thematic framework by sifting, sorting, coding and charting collected data [26]. This approach was adopted so as to provide findings and interpretations that are relevant to policy and also to provide pragmatic recommendations.

### Ethical considerations

This study sought and obtained ethics review and approval from the KEMRI scientific and ethics review committee.

## Findings

This section begins by presenting findings on the organizational structure of the hospitals, followed by findings on the extent of hospital autonomy in the five domains of the study’s conceptual framework namely strategic management, financial management, procurement, human resource management, and hospital administration.

### Hospital organizational structure

**Fig 1:**
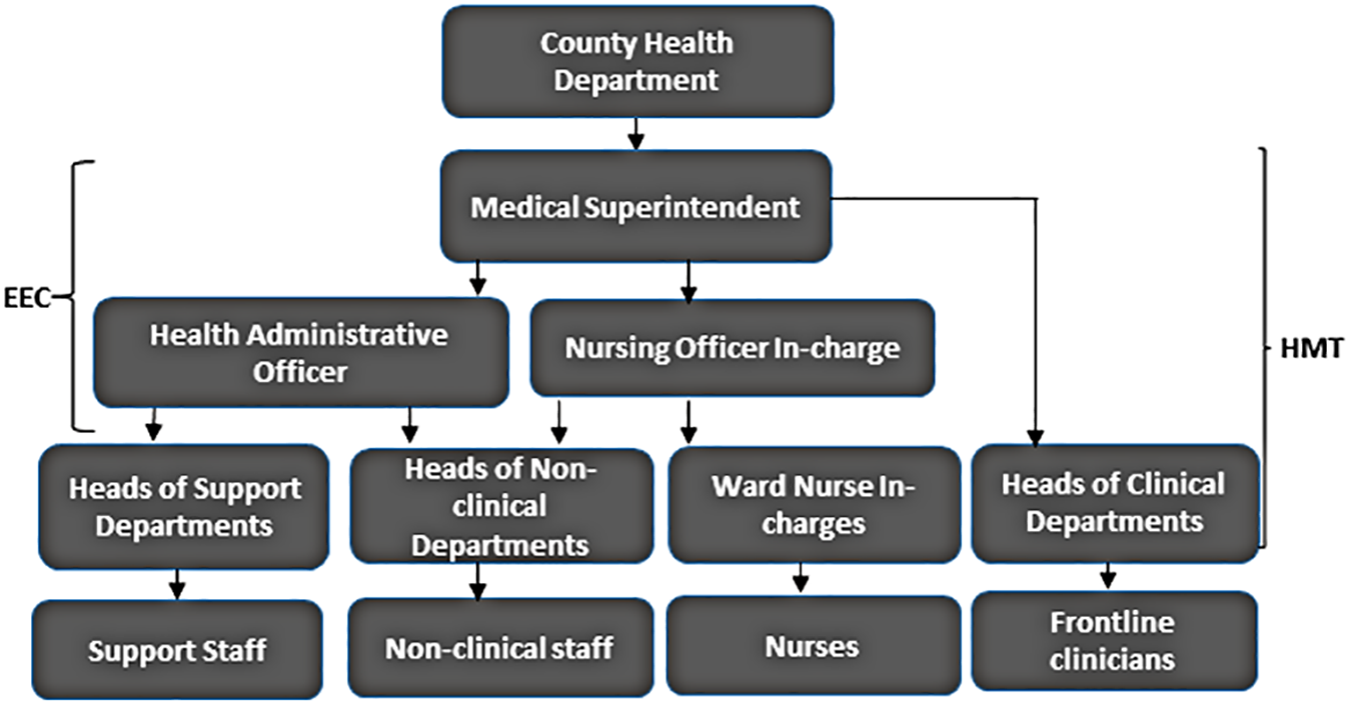
Hospital organogram.

The three case study hospitals did not have an official organogram. However, our observations and descriptions by the study respondents implied the existence of an organizational structure presented in Fig 1 below.

The hospitals were hierarchically organized into three layers. Frontline practitioners (such as medical doctors, pharmacists and nurses) form the first layer and are at the interface of service provision between the hospital system and the users of hospital services. These frontline workers are organized into service delivery units and departments that are headed by middle level managers. For example, clinical departments are headed by clinical specialists, while the various hospital wards, and the outpatient department are headed by nurse managers (in charges). The middle level managers in turn report to senior level managers. Senior level managers in the hospital included the medical superintendent (who was the chief executive officer of the hospital), the hospital administrative officer, and the hospital nursing officer in charge (hospital matron) who was assisted by a deputy matron. The hospital had two major management committees. The hospital management team (HMT) chaired by the hospital superintendent and comprised of hospital administrator, hospital matron and all departmental heads. The executive expenditure committee (EEC) was also chaired by the medical superintendent and comprised of hospital matron, hospital administrator, accountant and procurement officer. In the pre-devolution period, hospitals had a hospital management committee (HMC) that comprised of community representatives and provided oversight to hospital affairs[27]. Post-devolution, the HMC did not exist, rather, the county health department provided oversight to the hospital.

### Strategic management

The hospitals operated under the same mission and vision that had been developed by the hospital in the pre-devolution period. In the pre-devolution period, the hospital developed strategic/investment plans implemented by finances at their disposal, and 1 year annual work plans [27]. Under the devolution arrangements, the hospitals did not develop an autonomous strategic plan as they had low financial autonomy. Rather, hospital plans, together with all other health facilities in the county, were incorporated in the county department of healths’ 5-year strategic plan (2013–18) which addressed health priorities, resource mobilization, investments, and implementation arrangements among other issues.

“No we are not involved in making such long term plans. Plans are made by the county and we are expected to follow these plans. We dont really have an input in the planning process.” Senior manager, Hospital C

The hospitals retained the autonomy to develop annual work plans (AWP). However, as in the pre-devolution period, AWPs were heavily guided by the national government and hence did not confer meaningful autonomy to the hospitals [28]. In the post-devolution period, AWPs were developed, after compiling plans from each department, and forwarded to the county government for budgeting. However, since hospitals lacked control over finances, planned activities were often not budgeted for, which resulted in the non-implementation by hospitals.

“We still have the roles of developing the hospital annual work plans. But we do not implement them. You see if you make a plan, you also need resources to implement the plans. Since we dont have the resources, how can we implement the plans?” Senior Manager, Hospital B

“You see after devolution, developing annual work plans is like building a castle in the air. We dont know the amount [of funds] we are entitled to so the annual work plans are just wishes that we dont get to implement. That is our problem with hospital planning.” Senior manager, Hospital C

Low autonomy over hospital planning resulted in misalignment of priorities between the county and hospitals. This was thought to reduce the hospital’s responsiveness to its clients’ needs.

“The county health department does not understand our priorities. Instead of buying a lot of ambulances they should have hired more staff for us. Instead of building a mortuary they should have built for us another ward, or an extra theater for maternity” Senior manager, Hospital A

“We have a ward here it is called Bajaj [mechanized three wheeled taxi] because we get a lot of patients who have had accidents while riding these Bajaj’s. If you walk into the ward you will see many so we call this ward Bajaj ward. Because we have so many of these accidents, a priority would be to put up another Bajaj ward, but the county health department does not understand our priorities.” Senior manager, Hospital C

### Financial management

Pre-devolution, hospitals were financed through 4 main sources: 1) budget allocations from the national government 2) user fee revenues from users of hospital services 3) Reimbursement from the National Hospital Insurance Fund (NHIF), and 4) Donations from non-governmental organizations and other charitable donors [27]. Post-devolution, hospitals retained all the financing sources other than budget allocations from the national government. A number of the changes in the hospital financing arrangements that accompanied the devolution process resulted in reduced autonomy of hospitals over financial management. First, in the pre-devolution period, budget allocations from the national government were transferred to bank accounts operated by the hospital. Post-devolution, the hospitals did not receive budget allocations from either the national or county governments. Rather, the hospitals were required to place requests for needed goods and services which were then procured and paid directly the county government.

“We are not getting allocation, what we usually do is when we need something we need to do a requisition [to the department of health].” Senior manager, Hospital C

Second, in the pre-devolution period, the hospitals were allowed to open and operate a bank accounts. These account was used to manage all hospital funds. Under this arrangement, the hospitals collected and banked user fee revenues from users to the hospitals bank accounts. The signatories of the hospital bank accounts were hospital staff and representatives from the district treasury, which provided the hospital with the autonomy to access and use the funds collected from user fees. Post-devolution, the hospital bank account was operated jointly by representatives of the hospital and county government. The hospitals bank accounts had 2 signatories from the hospital and 2 from the county government who were mandatory signatories. The hospitals could only deposit funds but could not access and use deposited funds.

“So [before devolution], either for us [hospital and district] could authorize a transaction at the bank. On our [hospital] part, either the hospital administative officer or medical superintendent could authorize a transaction, while on their [district] part, either the district accountant or the deputy district accountant could authorize a transaction. But now both parties [hospital and county health department] have to sign, they are mandatory signatories.” Senior manager, Hospital B

Third, and related to the reduced autonomy over the ownership and control of hospital accounts, the autonomy of the hospitals over the management and use of user fee revenues was removed. In the pre-devolution period, the hospitals developed quarterly budgets that outlined their planned use of, among others, user fee revenues. The hospitals then submitted these budgets to the national government and obtained an authority to incur expenditure (AIE), which allowed the hospitals to use user fee revenues in line with their submitted budgets. Given that the hospitals operated their bank accounts, they had significant flexibility over access and use of user fee revenues. While hospitals could budget for, and had access to hospital revenues, hospital managers felt constrained by the line item budgeting guidelines [29]. However, post-devolution, the hospitals were no longer allowed to retain their user fee revenues and were expected to bank these revenues to the county revenue fund account, which was an account that managed revenues from all other revenue generating sectors in the county. The hospital was required to place requests for goods and services to county governments, which then directly procured them on behalf of the hospital.

“The hospital is just but a collection center, a collection point for the revenue systems of the County Government. This makes it difficult for us as a hospital because we also need to spend but we are not allowed. We are now required to make requests to the various county heads for approval so that we can be able to get items or commodities that we require.” Senior manager, hospital B

“For example there was a time we had an issue with fuel, if an ambulance doesn’t have fuel you can’t buy fuel for that ambulance because you are not allowed to spend user fee money directly at the hospital. We cannot sort out day to day maintenance issues, drug stock outs…we cannot respond to emergencies that require spending. This user fee issue has really affected us [the hospital].” Senior manager, Hospital A

This change was occasioned by a new public finance management law (The Public Finance Management Act 2012) which took away the hospitals’ freedom to retain, bank and spend collected revenues. The law required that all collected revenues had to be pooled to the County Revenue Fund (CRF) account. Revenues collected and deposited in the CRF were considered county revenues, and therefore the hospitals had lost all residual claims to collected revenues from user fees.

“With coming of devolution we have the PFM-Public Finance Management Act which doesn’t allow them now to just bank and get and use like what used to happen before.” County level manager

The reduced autonomy over financial management had a number of effects in the hospitals. First, reduced access and control over user fee revenues resulted in delays in the procurement of essential supplies.

“I wish they could give us the authority to incur expenditure like before so that we can avoid all these unnecessary delays. When we need to purchase something, we should be able to purchase it without all these delays.” Middle level manager, Hospital C

Second, hospital respondents reported that reduced autonomy over user fees resulted in reduced staff motivation due to inability of the hospital management to provide a favorable environment for efficient working.

“So for one to work effectively and efficiently they need to have everything that they need in order to give their services, but once you don’t have one or two things, once you don’t have the basic things that you are supposed to be having in order to give the services you also get demotivated.” Senior manager, Hospital A

“Each time we sit for our health management team meetings the same question arises, we collect a lot of user fee revenues, why can’t we use that money on this and that. And you see if you can’t use it even to at least do some maintenance within your departments then of course the staff who are working there they get demotivated. They wonder why are they bothering to help generate income if that same income is not coming back.” Senior manager, Hospital B

Third, it was reported that donor support had considerably reduced after devolution in all 3 hospitals. One of the reasons advanced for this was the reduced autonomy of the hospitals to deal directly with the donors.

“We can say, relating to donors is a bit difficult because the donors have to come through the head of the department [in the county government], but they cannot come directly like the way they used to come directly to the hospitals.” Senior manager, Hospital C

“In terms of donors, currently getting donors, it has really affected since when… for you to be able to get a donor, you also have to show what uhh the hospital intends to do because most of the donors usually do support part, and uhh expect that you also chip in so that it can be like a cost sharing sort of thing. But in our case now, it’s becoming difficult since in our case we can’t be able to support and we’ve not been allowed to make expenditures from the accounts.” Senior manager, Hospital B

To circumvent these challenges, hospitals reported subverting the law and illegally spending user fee revenues at source.

“We are forced to use money at source, which as you know is illegal. The problem is once we bank the money we cannot access it anymore. But we have immediate and pressing needs in the hospital which forces us to illegally use collected user fees at source.” Senior manager, Hospital A

“According to the county regulation we’re not supposed [to spend user fees at source]. . . . But now what do you do if you need fuel to transport a patient by ambulance? We just have to break the law and spend the user fees for such emergencies.” Senior manager, Hospital C

The county government had however recognized the limitations occasioned by this legal restriction and was in the process of exploring legislative options that would give the hospital more autonomy over financial management. For example, the county government had developed a Health Services Management bill that would create a legal framework for the hospital to increase autonomy over financial management. The law had however not been effected at the time of data collection.

“It required us to come up with a legal framework for them to be able to access the funds and we sat and we drafted the Health Services Management bill for Kilifi County and when it went to the cabinet, instead of leaving the cabinet to county assembly it found its way to the government printer which was a mistake, so that became a hitch again and therefore it had to go back to the assembly again. That has made the process long.” County level manager

“We have agreed the other way is for him [county minister for Finance] to give us the authority to open expenditure accounts for user fees then we will be able to collect, bank and then after that the money is swept into the county revenue fund to National Treasury and then it bounces back to expenditure account from where they will spend the money for purposes of auditing and monitoring. That is in process.” County level manager

### Procurement

In the pre-devolution arrangement, the hospitals had some autonomy in the procurement of goods and services. The case hospitals received supplies prepaid by the national government mainly from the Kenya Medical Supplies Agency (KEMSA), but also procured supplementary supplies directly from suppliers [28]. Hospitals complained that while they roles included procuring medicines, this role was restricted by the narrow choice of medicines in the essential medicines list prescribed by KEMSA [28]. Under devolution, the procurement autonomy of hospitals had worsened. Hospitals were no longer procurement entities, and were required to compile and submit their requirements to the county health department, which then procured and made payments on their behalf.

“The procruement process is not decentralized…it is centralized to the county health department level… I still cannot believe that we are still doing centralized procurement even when we are supposed to be devolved.” Middle level manager, Hospital A

“Sometimes you get a letter that you have been appointed to the procurement committee, but it is meaningless. It is meaningless because procurement is done by the county health department so here in the committee we just talk and talk but we have no real authority.” Middle level manager, Hospital A

The procurement process took long as there was need for a number of approvals, such as for availability of funds to procure and authorization for payments among others, at the county headquarters. While such approvals were present pre-devolution, they were swiftly done at the district headquarters which were closer and less busy compared to the county headquarters which now dealt with all former districts. Further, payments for suppliers for all county departments was made through a single pool in the county treasury. Therefore, payment time depended on perceived priorities and influence to pay that the person responsible for payments had. Due to these, most respondents across the 3 hospitals felt that the procurement process had become too bureaucratic and slow.

“Before [devolution] the process of procurement used to be very swift. That is, there were no bureaucracy. Now, the process is much slower and bureacractic. We experience serious delays in procurement.” Middle level manager, Hospital B

“When you place a request for something, you find that the procurement process is slow and cumbersome …it’s not as fast as it used to be.” Senior manager, Hospital C

This change had a number of implications. First, there was a delay in delivery of supplies at times which affected service delivery.

“There is just so much unnecessary delays. So something which you could have maybe gotten within a span of say a month now can take you up to three or four months.” Senior manager, Hospital B

“The process is slow that sometimes it doesn’t even make sense…you’ll have taken two months to order the supplies for one month, you get the scenario.” Senior Manager, Hospital A

Second, most suppliers had become unwilling and had halted supplies to the hospitals because of delayed payments. This lead to shortages of essential medical supplies to hospitals.

“So, our fuel supplier, remember he is the same guy who is supplying [fuel for] ambulances, utility vehicles and all the vehicles that we have in the hospital. He gave us some credit for about a month, after a month without any payment he stopped providing us with fuel. In any case, he has the rights, he has supplied, he has not being paid, he has given credit, we’ve exceeded the credit limit and the credit period, then he has all the rights to stop providing fuel. But then you see this is a very essential commodity, we need that fuel to run the ambulances. We need the same fuel maybe for generators.” Senior manager, Hospital A

“Because of the lengthy and slow process, payments to suppliers are delayed. The suppliers has also to go through the same middle man [county governement] before they get their money The suppliers are no longer keen on supplying to the hospital anymore.” Senior manager, Hospital B

### Human resource management

Under the devolved system of government, the human resource function in the health sector had been transferred from the national government to the county government. As with the pre-devolution arrangements, the hospitals had professional staff (such as doctors, pharmacists, nurses, administrators) who had permanent employment contracts, and support staff (such as driver, cleaners, security guards) who had short term employment contracts [29]. While hospitals had autonomy over support staff, they complained of their lack of autonomy over professional staff, and expressed a desire to have greater control over this later group [29]. Hospital’s lack of autonomy over human resource was therefore worsened under devolution. Professional staff were recruited, remunerated and deployed to the hospital by the County Public Service Board (CPSB) under the post-devolution arrangements. The significant change with regard to the hospital over human resources was in the management of support staff (casual workers). The recruitment, remuneration and management of support staff was transferred from hospitals to the CPSB. This meant that hospitals had now lost autonomy over all (professional and support) staff.

“The hospital had the mandate to employ as they wish. Now they are not because laws have changed and the county now has a County Public Service Board, which is a representation of the Public Service Commission at the national level.” County level manager

The reduced autonomy of the hospital over the management of support staff had a number of effects both positive and negative. First, recentralization of recruitment of causals had taken off the pressures of hiring and remunerating from hospital managers.

“One of the advantages maybe would be the fact that you have a peace of mind maybe, we don’t physically touch the cash [for paying casuals] so just maybe that peace of mind.” Senior manager, Hospital B

“We dont have that pressure people applying to us, people following us like am giving you my application letters you push it for me.” Senior manager, Hospital A

Second, it was reported that the casuals now enjoyed better perks compared to when the hospitals were responsible for their remuneration.

“So, the county took over the payment of casuals sawa [okay?], I can say it is not bad because after the county took over in fact the perks improved. They are now getting better perks that we could not pay with the hospital user fees.” Senior, Manager Hospital A

Third, some managers felt that their lack of autonomy over recruitment and payment of support staff resulted in increased indiscipline and insubordination by these category of hospital staff.

“The casuals are a bit reluctant because they know that the medical superintendent has no power to fire them.” Senior manager, Hospital C

“Cases of indiscipline have actually increased. Yes some [casuals] are insubordinate. This is because they have been brought here by “big fish” or political god fathers, so you cant touch them.” Middle level manager, Hospital A

Fourth, there had been concerns of hiring of unqualified persons, nepotism and tribalism in recruitments, which was likely to negatively affect service delivery.

“Some of the casuals that are assigned here don’t even know how to read or write but you cannot say no. You find this person is weak and will not even be able to do his work well, but then you have to keep that person because he or she has been brought by a county boss, now that is how life is here.” Senior manager, Hospital C

“You may find there is someone who already has been trained has the proper qualification and that’s the person we would like to be hired but is not the person who is hired. Through the system we don’t know what happens but you end up getting someone who may not be as qualified and then you have to you just have to put up with them. And the problem also with that is you know people some people are hired because of connections connection in terms of family ties and so on. So it has become problematic, others are hired because or ethnicity. It’s wrong to say but there are some ethnic influences in to play.” Senior manager, Hospital B

### Hospital administration

Overall, hospital respondents felt that devolution had resulted in reduced autonomy of the hospital over key functions.

“When you compare before devolution and after devolution…it seems that after devolution things have become worse, it is like things are not decentralised but rather they have become centralised. Things are supposed to be decentralised isn’t it? Things are supposed to be decentralised to the ground, now they are centralised.” Senior manager, Hospital A

The reduction of hospital autonomy over key functions appeared to affect the administration of the hospital in a number of ways. First, reduced autonomy appeared to weaken the leadership and management structures across the 3 hospitals. For example, while previously the hospitals had two functional decision making committees, the EEC and HMT, both committees had become dysfunctional as a result of reduced autonomy. Although the HMT was still in place with the same structure and composition as before devolution, its powers, especially with regard to financial management, had been reduced. Decisions made by the HMT required the approval of the county department of health.

“Devolution has interefered with these management committees. Take for example the HMT, their work was to prepare hospital budgets. But how can you budget without a resource envelope? it would be wishful budget, because we have no control over resources anymore. All the committees has become domant…you could not be having committees without power and authority.” Middle level manager, Hospital A

“These meetings, so many of them, are meaningless, because of lack of resources. So, if you were talking about resource, resource, resource, and there is no resource, you will just end the meetings because now, it will boring, what are you meeting on about? Because everything is being decided by the county health department. They [county health department] are pooling all resources together. We no longer have access to user fee revenues.” Senior Manager, Hospital B

Further, respondents felt that the reduction of the powers, and hence control over resources, of the office of the medical superintendent over decisions and resources made the position less attractive to hospital staff. As a result, hospital staff were unwilling to take up this position. This was evidenced by the high turn-over, and the difficulty in filling this position. At the time of collecting data, one of the case hospitals had not had a medical superintendent for 2 years, while the other experienced a high turnover of medical superintendents. Hospital leadership had therefore been weakened by reduced autonomy.

“The powers have reduced, they are just here, they just deal with papers, maybe before they used to handle the accounts and everything maybe they are[were] getting something small, so of late now they are just… they want to be… at least given something to be in this office that is what I have learned.” Middle level manager, Hospital C

“They are demoralized, when they are asked to come and sit here, like there is one who has just resigned.” Senior manager, Hospital A

”We have advertised the position but nobody responds. It’s not easy these days to get a hospital medical superintendent.” County level manager

Increased bureacracy resulted from the transfer of hospital committee functions to the county department level. This was thought to result in delays in decision making that negatively impacted on the quality of services provide by the hospitals.

“It affects quality of services a lot. . . .quality of services have worsened and now people are getting poor quality care. For example, if a drug is out of stock, the HMT would sit and make an emergency or supplimentary budget and buy the drugs. But now you we have to order through the county and wait… it takes very long, so the quality of services that we give in the hospital now has detoriorated.” Senior Manager, Hospital B

“A major challenge with the increased bureacracy is delays in payment of suppliers. Most of our suppliers are no longer willing to supply us since have pending payments and debts. This has really affected service delivery. If the hospital does not have the commodities or tools required to provide care what do we do? it means that the quality of care offered to clients will not be good.” Senior manager, Hospital C

Second, reduced autonomy had weakened community’s involvement in hospital decision making. In the pre-devolution period, the channels for community involvement in hospital affairs included suggestion boxes and a hospital management committee (HMC) [30]. The HMC comprised of community representatives and provided oversight to the hospital management. This however did not work so well because there were concerns about the legitimacy and fairness of the process for selecting community representatives to the HMC, and the empowerment of these HMC members [30]. Even though these community involvement channels had not changed after devolution, as mentioned earlier, the HMC had not been in place after devolution. This had two major implications. First, hospitals completely lacked oversight of their operations and avenues for community participation in decision making in the hospital. Second, hospitals lacked a platform to engage stakeholders as this was a role played by the HMC. While this platform was weak pre-devolution, it was completely non-existent post-devolution.

“You see the HMC in the past used to help us to oversee some of the decisions we would make, because the HMC would consist of individuals from the community. So it would help us at least steer us in the right direction on some of the decisions that we were making, it used to help us also to connect with the community. So I would say right now as a hospital at times we are not that connected to the community and maybe some decisions that could be made may not be in line with the needs of the community.” Senior manager, hospital B

“The decisions we make now are one sided…they come from people inside the hospital. You also need someone to have a look from outside and tell you, you know there is this and this. So I think that is where without the HMC that’s where we are lacking guidance from the community.” Senior manager, hospital A

County respondents reported that, even though the county government supported the establishment of hospital management committees, the county lacked a legal framework to appoint members to the committee.

”We have names [of HMC members] but we can’t gazette them before the legal framework tells who should gazette. We would like all hospitals to have HMCs to oversee their activities, but first we need a legal framework, which is lacking.” County level manager

## Discussion

This is the first study in Kenya, and one of a few in LMICs that examines the effect of decentralization reform on hospital autonomy and functioning. While it is generally expected that decentralization reforms increase decision space and autonomy at local levels [18], our findings show that this is not always the case. Rather, it depends on the specific level of the health system one is interested in, and how decentralization has been designed and operationalized in a particular context. Before 2013, the Kenyan healthcare system was highly decentralized in the form of deconcentration. Under this arrangement hospitals had some level of autonomy over management functions even through there was still scope for increasing their autonomy [28]. Our overarching finding is that after 2013, when the devolution form of decentralization was introduced, hospitals experienced a significant reduction in autonomy over the 5 key domains of hospital functions because of the transfer of key functions from hospitals to county health departments. Hospitals are therefore experiencing ‘recentralization within decentralization’. Our findings are similar to the Philippines experience, where the vacuum of regulations, management systems and administrative culture that resulted from loss of a national bureaucracy was rapidly filled by the local government political authority [31].

Reduced autonomy of hospitals, and the challenges faced as a result was occasioned by the institutional design of devolution, which had reassigned the authority and power that sub-county structures such as hospitals had pre-devolution to the county level. For example, while previously hospitals were allowed to employ non-professional staff, this role has been transferred to the newly formed public service boards. However, the loss of financial autonomy perhaps highlights the role that actors and their interests in the devolution process. The explanation given by study respondents for the lack of financial autonomy was the fact that the public finance management act (2012) required that all revenues collected within the county are banked and managed from one county revenue fund account, which is under the control of the county treasury. However, a keen examination of devolution laws reveals that, while the public finance management act requires that counties operate one county revenue account, the law also provides for county governments to develop bylaws that will allow them to give financial autonomy to units such as healthcare facilities. However, vested interests have led to all sub-county units, including healthcare facilities losing residual claims over the resources they generate. County leaders, who now have the power and control over resources are unlikely to willingly give up these privilege back to the hospitals. For instance, while Kilifi county had developed a draft law that would allow hospitals to retain and use revenues collected at source, this law had not been formally adopted for over 2 years because of an impasse at the county assembly. Even though we did not investigate this, it is very likely that power dynamics have and continue to play a role in the delay of enactment of enabling legal frameworks.

The reduced autonomy of hospitals over key functions had a number of effects. First, the management and leadership of the hospitals was weakened. It has been shown elsewhere that increasing hospital autonomy results in the strengthening of hospital leadership and management because of the need to manage more functions [32]. Our findings show that when autonomy is reduced, the opposite effect can be observed. When functions are removed from hospitals without clear realignment of the roles of management structures, these structures become weakened. Further, hospital staff are less enthusiastic about taking on leadership and management roles that have reduced power or control over resources. Second, reduced autonomy weakened the external accountability of hospitals by removing a key platform for community participation, the hospital management committee. While the HMCs were weakened pre-devolution, they were non-existent post-devolution. This puts to question the legitimacy and responsiveness of the management decisions of the hospitals. Third, reduced hospital autonomy is shown to introduce inefficiencies in the hospitals due to increased bureaucratic delays, poor staff motivation, staff insubordination and political involvement in staff recruitment, and stock outs of essential supplies. For instance, while in the pre-devolution period, delivery delays by the KEMSA were offset by local procurement from private pharmaceutical distributors by hospitals [28], this option was not available post-devolution. The finding on reduced staff motivation is similar to what was observed in Uganda where limited role of staff in control of resources meant inability for them to make decisions on needs and priorities which limited their motivation to improve performance [33]. These findings are also similar to other settings such as Fiji, where after decentralization the health facilities had little or no control over human resource management as there was a high degree of centralization of the civil services which were managed by the Public Service Commission [16]. In Philippines, perceived political recruitments were reported in the first year of devolution due to increased influence of local governments and reduced regulatory control by the national department of health [31]. This resonates with findings in other setting where increased autonomy was shown to increase efficiencies [34,35]. Fourth, stock out and delays in delivery of essential supplies, and the lack of alignment of county and hospital priorities compromised the quality of services delivered by the hospitals. This also reinforces the observations that increased hospital autonomy is associated in increased quality of services [34,35].

## Conclusion

It is clear that there is a need to increase the autonomy of county hospitals in Kenya. To do this, county governments would need to review the institutional framework for the governance of hospitals with the aim of providing for greater autonomy and decision space of county hospitals. First, a legal framework should be put in place for the creation of hospital management committees that will provide oversight to hospital management and provide an avenue for community participation. Second, the county governments should exercise the rights given by the public finance management act of Kenya, develop, and implement by laws that provide for sub-county units such as hospitals to have residual rights over the revenues that they generate. Third, county hospitals should be given back control of the management of key functions such as procurement and human resource management. Fourth, the management and leadership structures of county governments should be realigned to the devolved system and strengthened by ensuing there is clarity of roles and responsibility as well as authority. Managers need decision space and the flexibility to effectively discharge their roles and to remain motivated in these roles.

Our study has a number of limitations. First, we restricted our interviews to hospital managers because we were specifically interested in management functions. Including frontline practitioners and community representatives could potentially enrich our findings. Second, the study took place in one county, and hence the inability to generalize findings to other counties in Kenya. This not-withstanding, this study, provides useful insights into the potential effects of decentralization reforms on the autonomy and functioning of hospitals, insights that can be considered and tested in comparable settings [36].
